# Fixed Drug Eruption Caused by Garenoxacin: A Case Report and Literature Review

**DOI:** 10.7759/cureus.48596

**Published:** 2023-11-10

**Authors:** Natsumi Hara, Natsuko Saito-Sasaki, Yu Sawada

**Affiliations:** 1 Dermatology, University of Occupational and Environmental Health, Kitakyushu, JPN

**Keywords:** literature review, patch testing, garenoxacin, case report, drug eruption

## Abstract

A new quinolone antibiotic called garenoxacin was developed in Japan. Garenoxacin is known to produce cutaneous adverse effects, particularly fixed drug eruption in Japan, despite several reports of cutaneous adverse events in English-language literature. However, English-language literature has not yet reported that fixed drug eruption is a common clinical manifestation of garenoxacin-induced drug eruption. In this article, we present a case of multiple fixed drug eruptions and review the literature on case reports of drug eruptions caused by garenoxacin.

## Introduction

Garenoxacin is a new quinolone antibiotic developed in Japan [1]. As a unique characteristic, a fluorine molecule at the C-6 position is absent in the new quinolone garenoxacin. Garenoxacin exhibits strong efficacy against many different bacterial infections [1]. Despite the representative clinical manifestation of garenoxacin-induced drug eruption being recognized as fixed drug eruption in Japanese literature, the detailed characteristics have not been reported in the English-language literature [2,3]. Here, we report a case of multiple fixed drug eruptions in addition to the previously published English and Japanese literature on garenoxacin-related drug eruptions.

## Case presentation

**Figure 1:**
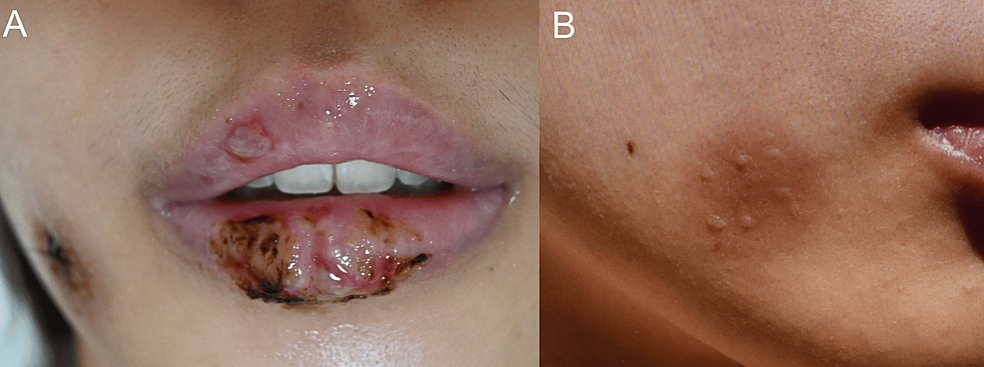
Clinical manifestation and examinations. (A) Clinical manifestation of skin eruptions following garenoxacin. (B) Clinical manifestation of the first eruption in the past following garenoxacin.

A 25-year-old woman who had a sore throat was treated with acetaminophen, desloratadine, montelukast sodium, garenoxacin, and L-carbocysteine. A few hours later, she noticed erythema on the cheeks and lips (Figure 1A), as well as mild mucosal erosion that was making it difficult to swallow food. She was referred to our department for the evaluation of her skin eruption. She had previously experienced skin eruption with small blisters after using garenoxacin (Figure 1B), suggesting that this may have been a culprit for her skin eruption.

A physical examination revealed that a scaly pigmented round-form macule with black clasts was located on her right cheek (Figure 1A), which was similar to her previous reaction that was noted after taking garenoxacin. Mucosal erosions were associated with crusts and pustules on the lip, and the buccal mucosal cavity also developed multiple aphthae and ulcers.

**Figure 2:**
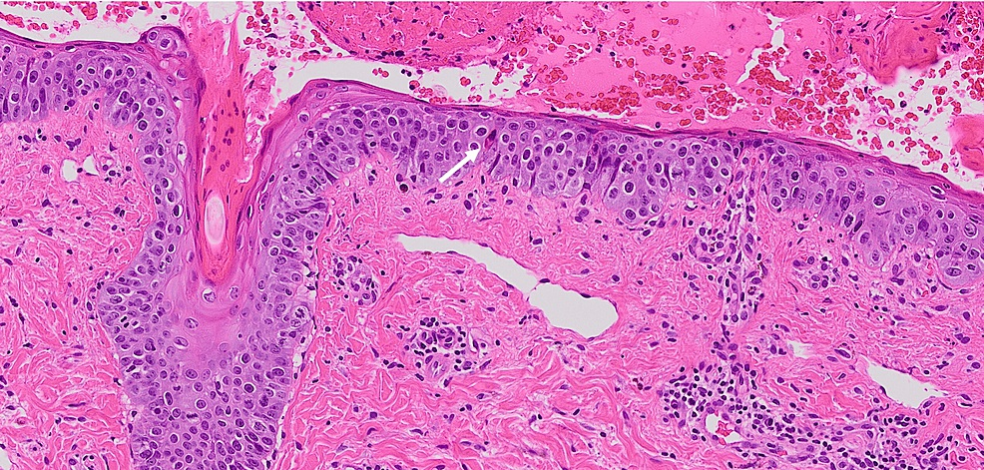
Histological examination. A skin biopsy showing dyskeratotic keratinocytes in the epidermis and infiltrated lymphocytes into the epidermis and dermis. The white arrow indicates the dyskeratotic keratinocyte.

A skin biopsy taken from the lip lesion revealed dyskeratotic keratinocytes in the epidermis and infiltrated lymphocytes into the epidermis and dermis (Figure 2). Based on the clinical manifestation and laboratory examination, we diagnosed her skin eruption as a fixed drug eruption.

**Figure 3:**
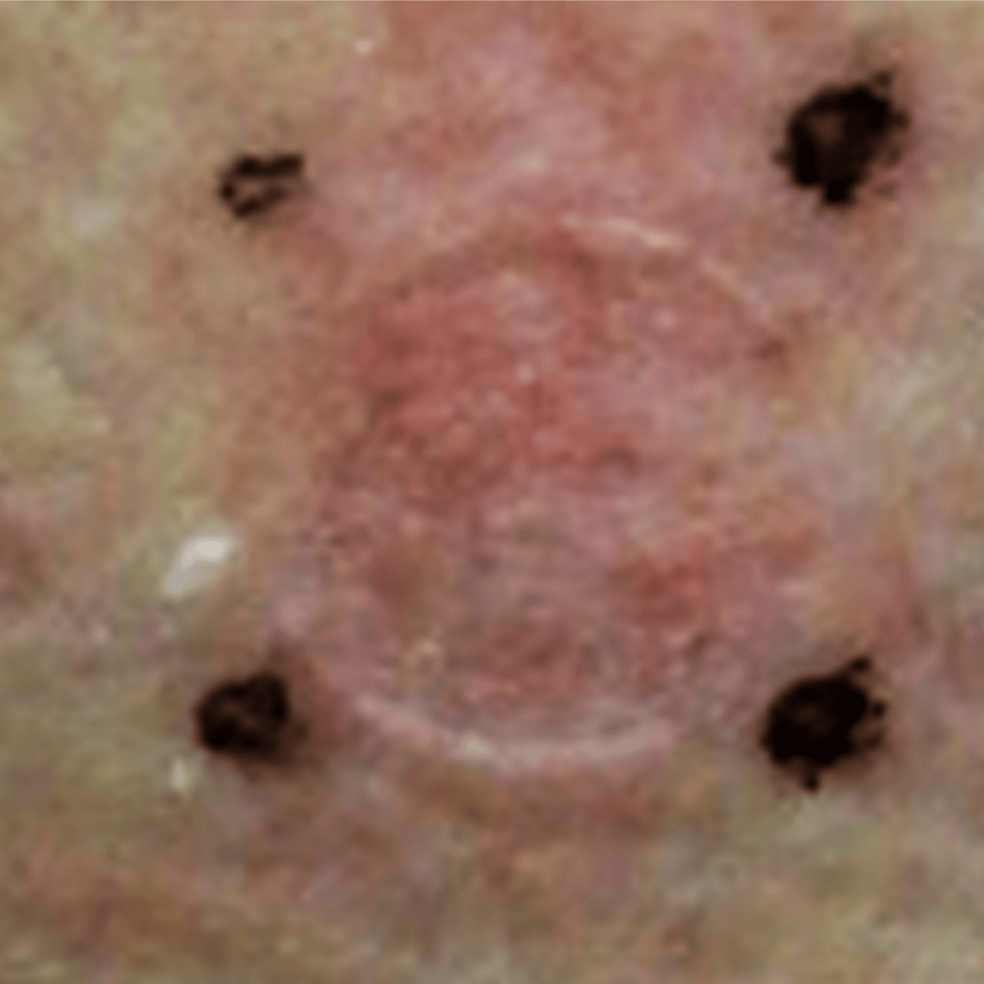
Patch testing. Patch testing using garenoxacin showed a positive reaction.

To explore the causative agents, we first conducted lymphocyte stimulation tests because the multiple fixed drug eruption has been known as useful in identifying the causative agents; however, all the drugs at a concentration of 10% showed negative results. We then conducted patch testing and 10% of garenoxacin showed a positive reaction 48 hours after the patch testing (Figure 3). The previous history of skin eruption following garenoxacin intake was also confirmed as a recurrence of skin eruption at this time with a positive patch test. In addition, the challenge test is the most conclusive test to identify the culprit; however, we could not obtain consent to conduct the challenge test using garenoxacin because the re-occurrence of mucosal skin lesions might be a risk for the patient. Therefore, we concluded garenoxacin is the causative agent of her fixed drug eruption.

Prednisolone 25 mg per day was administrated for three days with the discontinuation of garenoxacin. Mucosal erosions and skin erythema rapidly improved, and the prednisolone dose was decreased to 20 mg per day for seven days. One month after the start of treatment, steroids were tapered off. The patient was discharged from the hospital with a favorable clinical course without any recurrence of her skin eruption.

## Discussion

Fixed drug eruption is recognized as repeatedly erythematous pigmented plaques following the same causative agents between 30 minutes and eight hours (on average two hours). Blisters occasionally occur on fixed drug eruptions [4] and can also spread widely and cause systemic symptoms including fever [5]. These clinical features might resemble Stevens-Johnson syndrome or toxic epidermal necrolysis.

Garenoxacin is a representative causative drug causing fixed drug eruption. To clarify the characteristics of garenoxacin-related drug eruption, we reviewed all English and Japanese reported cases of drug eruption caused by garenoxacin (Table 1) [2,3,6-13]. In total, 11 cases have been reported, including our case. The male-to-female ratio in the studies was 4:7, and the mean age was 42.5 years. There are several types of drug eruptions. The most common types were multiple fixed drug eruption (eight cases), followed by maculopapular type (two cases), and drug-induced hypersensitivity syndrome (one case). Several examinations for the identification of causative drugs were tried in previous studies. The oral challenge test was the most reliable examination and showed a 100% positive reaction in five cases. Patch testing showed a positive reaction to garenoxacin in 57.2% of cases. On the contrary, a lymphocyte stimulation test had a negative result in all cases without any specific findings on blood testing. As our case showed mucosal lesions and we could not obtain consent to conduct a challenge test, lymphocyte stimulation test and patch testing were conducted. All these cases exhibited favorable clinical behavior without recurrence of skin eruption after the discontinuation of the drug.

**Table 1:** A literature review of case reports of garenoxacin-associated drug eruptions.

Authors	Age/Gender	Drug eruption type	Interval	Location	Identification of causative drug
Matsumoto et al. [6]	40/Male	Fixed drug eruption	6 days	Hand and foot	Patch testing: positive; oral challenge test: positive
Harada et al. [7]	64/Male	Fixed drug eruption	3 days	Elbow and thigh	Patch testing: negative; oral challenge test: positive
Yamamoto et al. [8]	63/Male	Fixed drug eruption	1 day	Oral mucosa and penis	Patch testing: negative; oral challenge test: positive
Takimoto et al. [9]	37/Female	Fixed drug eruption	1 hour	Lip, neck, arm, and thigh	Patch testing: positive
Mimura et al. [10]	26/Male	Fixed drug eruption	A few hours	Conjunctiva (eye), lip, and foot	Lymphocyte stimulation test: negative; patch testing: negative; oral challenge test: positive
Ohara et al. [11]	31/Female	Maculopapular type	1 day	Entire body	Lymphocyte stimulation test: negative; patch testing: negative; oral challenge test: positive
Sato et al. [12]	43/Female	Fixed drug eruption	1 day	Oral mucosa, finger, abdomen, and lower back	Oral challenge test: positive
Takenoshita et al. [13]	33/Female	Drug-induced hypersensitivity syndrome	26 days	Entire body	Lymphocyte stimulation test: positive
Oda et al. [2]	72/Female	Maculopapular type	14 days	Entire body	Patch testing: positive
Miyake et al. [3]	33/female	Fixed drug eruption	2 days	Chest and buttocks	Patch testing: positive
Our case	25/Female	Fixed drug eruption	A few hours	Lip, oral mucosa, and cheek	Lymphocyte stimulation test: negative; patch testing: positive

## Conclusions

Garenoxacin was first developed in Japan, and garenoxacin-related drug eruption has been mainly reported in Japan. The characteristics of garenoxacin-related drug eruption are specific and fixed drug eruption is a typical cutaneous adverse reaction. We reported a representative case and discussed the previously reported cases mainly described in the Japanese literature. Our literature review will provide useful information for clinicians to obtain a better understanding of fixed drug eruption as a representative garenoxacin-related drug eruption.
